# Characterization of fungal *RTG2* genes in retrograde signaling of *Saccharomyces cerevisiae*

**DOI:** 10.1111/1567-1364.12055

**Published:** 2013-06-20

**Authors:** Ercan Selçuk Ünlü, Lakshmi Narayanan, Donna M Gordon

**Affiliations:** Department of Biological Sciences, Mississippi State UniversityMississippi State, MS, USA; Department of Chemistry, Abant Izzet Baysal UniversityBolu, Turkey

**Keywords:** Mitochondria/nuclear signaling, glutamate auxotrophy, citrate synthase

## Abstract

Changes in the functional status of mitochondria result in the transcriptional activation of a subset of nuclear-encoded genes in a process referred to as retrograde signaling. In *Saccharomyces cerevisiae,* this molecular link between mitochondria and the nuclear genome is controlled by three key signaling proteins: Rtg1p, Rtg2p, and Rtg3p. Although the retrograde signaling response has been well characterized in *S. cerevisiae*, very little is known about this pathway in other fungi. In this study, we selected four species having uncharacterized open reading frames (ORFs) with more than 66% amino acid identity to Rtg2p for further analysis. To determine whether these putative *RTG2* ORFs encoded bona fide regulators of retrograde signaling, we tested their ability to complement the defects associated with the *S. cerevisiae rtg2Δ* mutant. Specifically, we tested for complementation of citrate synthase (*CIT2*) and aconitase (*ACO1*) at the transcript and protein levels, glutamate auxotrophy, and changes in the interaction between Rtg2p and the negative regulator Mks1p. Our findings show that all four Rtg2p homologs are functional upon activation of retrograde signaling, although their degree of complementation varied. In addition, all Rtg2p homologs showed a marked reduction in Mks1p binding, which may contribute to their altered responses to retrograde signaling.

## Introduction

Functional mitochondria are integral to the survival of eukaryotic cells. Therefore, it is not surprising that cells have evolved an intracellular signaling pathway that responds to mitochondrial dysfunction. In yeast, this pathway is referred to as ‘mitochondrial retrograde signaling’ and describes the signaling pathway between mitochondria and the nuclear genome. Functional abnormalities in mitochondria activate the yeast retrograde signaling pathway through the cytosolic Rtg1p, Rtg3p, and Rtg2p proteins. The decreased membrane potential associated with dysfunctional mitochondria leads to activation of cytosolic Rtg2p, which results in the dephosphorylation of the Rtg1p-Rtg3p heterodimer activating the complex and causing its translocation from the cytosol into the nucleus (Jia *et al*., 1997; Miceli *et al*., 2012). Nuclear-localized Rtg1p-Rtg3p acts as a transcription factor, upregulating genes whose products function to compensate for the loss of mitochondrial activity including peroxisomal citrate synthase (*CIT2*) and proteins involved in the tricarboxylic acid cycle (Liao *et al*., 1991; Liao & Butow, 1993; Burns *et al*., 1994; Jia *et al*., 1997).

Although Rtg2p is central to retrograde signaling, Mks1p is key to the pathway’s regulation. When hypophosphorylated, Mks1p preferentially binds to Rtg2p, which leads to the dephosphorylation and nuclear translocation of Rtg1p/Rtg3p followed by activation of target gene transcription. On the other hand, the hyperphosphorylated form of Mks1p is complexed with Bmh1/2p where it functions to negatively regulate retrograde signaling by blocking Rtg1p/Rtg3p translocation. When not associated with Rtg2p or Bmh1p, Mks1p is polyubiquitinated in an SCF^Grr1^-dependent manner and targeted for degradation by the proteosome (Liu *et al*., 2005).

Yeast mitochondrial retrograde signaling components are conserved among a small range of yeast species (Liu & Butow, 2006). In this study, we aimed to characterize components of the retrograde signaling pathway present in other yeast. Key players in retrograde communication include Rtg1p, Rtg2p, and Rtg3p, and homologs of these genes have been identified in four distantly related yeast including *Candida glabrata*, *Ashbya gossypii*, *Kluyveromyces lactis*, and *Vanderwaltozyma polyspora*. In this work, we have focused primarily on characterizing Rtg2p, the master regulator of retrograde signaling. Using 66% amino acid identity as the cutoff point, we have identified putative *RTG2* genes from *C. glabrata*, *A. gossypii*, *K. lactis*, and *V. polyspora* and tested their ability to functionally complement a *Saccharomyces cerevisiae rtg2* deletion mutant. Measured complementation parameters included glutamate auxotrophy, *CIT2* and *ACO1* transcript levels, Cit2p and Aco1p protein levels, and the dynamic interplay between Rtg2p and Mks1p and Mks1p and Bmh1p. All *RTG2* homologs were able to rescue glutamate auxotrophy; however, not all were able to activate Cit2p and Aco1p expression, and all homologs showed a reduced interaction between Mks1p and Rtg2p.

## Materials and methods

### Strains and growth conditions

A list of plasmids used in this study are presented in Supporting Information, Table S1. The open reading frames (ORFs) for *RTG2* homologs were generated by PCR amplification using the appropriate genomic DNA as a template. The resulting DNA fragments were cloned into the NotI and BglII sites of YCplac22- and pRS414-based plasmids to generate *GPD* and *RTG2* promoter-driven expression of carboxy-terminal HA_3_ epitope-tagged *RTG2* ORFs, respectively. All constructs were confirmed by sequencing before use. Standard yeast genetic protocols were used to construct all strains that are derivatives of W303 (*leu2-3-12 trp1-1 can1-100 ura3-1 ade2-1 his3-11,15*) (Guthrie & Fink, 2002). Unless otherwise specified, all strains were grown at 30 °C in -Tryptophan synthetic dropout media composed of 0.67% Bacto-yeast nitrogen base (with ammonium sulfate), 2% dextrose, and supplemented with a modified Brent dropout mixture (BSM) lacking tryptophan (Saghbini *et al*., 2001). Supplements that differed significantly from the BSM standard were included at the final concentrations listed: 67 mg/L l-aspartic acid, 20 mg/L l-cysteine, 30 mg/L l-isoleucine, 80 mg/L l-leucine, 20 mg/L l-proline, 20 mg/L l-serine, 67 mg/L l-threonine, 67 mg/L l-valine. For ‘plus glutamate’ growth conditions, glutamate was provided at 100 mg/L as l-glutamic acid; otherwise, glutamic acid was also omitted from the modified BSM, and the media referred to as -Tryptophan -Glutamate.

For comparing growth under plus and minus glutamate conditions, cells were grown overnight in -Tryptophan synthetic dropout media at 30 °C with shaking. The next morning, cells were isolated by centrifugation (4000 ***g*** for 10 min), washed with sterile water, and resuspended in -Tryptophan synthetic drop out media or -Tryptophan -Glutamate synthetic dropout media to a density of 0.1 OD_600_. For all experiments, cells were grown to 0.5–6 OD_600_ before processing, which corresponded to 6–7 h of growth.

### Generating *RTG2* shuffle strains

Given that haploid *rtg2* deletion mutants exhibit altered fitness and chemical sensitivities, all functional studies were carried out using an *rtg2* shuffle strain (http://chemogenomics.med.utoronto.ca/fitdb/fitdb.cgi). The *rtg2Δ::kanMX6* shuffle strain was generated as described (Sikorski & Boeke, 1991). Briefly, the entire *RTG2* ORF was replaced with the *Escherichia coli* kanamycin-resistance marker in diploid yeast by homologous recombination (Longtine *et al*., 1998). Correct integration was confirmed by genomic PCR analysis, and the resulting diploid strain transformed with a *URA3-*marked plasmid (pRS416) containing the 1-kb *RTG2* promoter followed by the *RTG2* ORF. The resulting diploid was sporulated, and *rtg2Δ::kanMX6* haploid mutants containing the *URA3* covering plasmid identified by growth on selective plates.

Plasmids containing the ORFs of *RTG2* homologs were individually transformed into the *rtg2Δ* shuffle strain using a standard yeast transformation protocol (Schiestl & Gietz, 1989), with strains selected on synthetic dropout medium lacking uracil and tryptophan. Before use, covered haploid strains were grown in the presence of 5-fluoro-orotic acid (5-FOA) for 3 days to select against the *URA3-*marked *RTG2* plasmid.

Strains deleted for *MKS1* or carrying epitope-tagged versions of *CIT2*, *MKS1*, or *BMH1* were generated using a similar integration-based protocol. Strains containing multiple tags or gene deletions were generated by crossing the appropriate haploid strains followed by sporulation and tetrad dissection.

### Protein preparation and Western blot analysis

Total cellular protein samples were extracted by alkaline lysis followed by TCA precipitation as described (Riezman *et al*., 1993). Samples were solubilized in SDS-PAGE loading buffer by bath sonication. Unless otherwise stated, an amount of protein extract equivalent to 0.1 OD_600_ of original cell culture was used for Western blot analysis.

Protein samples were separated by SDS-PAGE, transferred to nitrocellulose, and stained with amido black to visualize protein standards and confirm equal loading. Proteins were detected using primary antibodies at dilutions suggested by the manufacturer (Covance) and HRP-conjugated secondary antibodies (GE Healthcare). The Pierce SuperSignal West Femto Chemiluminescence system (Thermo Scientific) was used for detection.

### Co-immunoprecipitation

2 × 10^7^ cells from exponentially growing cultures were harvested by centrifugation, and the cell pellet resuspended in 0.1 M Tris-SO_4_ (pH 9.4), 10 mM DTT. After 15 min on ice, cells were treated with zymolase (1 mg zymolase g^−1^ cell pellet) for 25 min at 30 °C to generate spheroplasts. Spheroplasts were lysed in RIPA buffer (25 mM Tris-HCl (pH 7.4), 150 mM NaCl, 1% NP-40, 1% sodium deoxycholate, 0.1% SDS) with half volume of acid-washed glass beads by five cycles of 30-s vortexing followed by 45-s incubation on ice. Cellular debris was removed by centrifugation at 20 000 ***g*** for 15 min at 4 °C. The resulting supernatant was transferred to a new tube, prewashed EZview™ Red anti-c-Myc or anti-FLAG affinity matrix (Sigma-Aldrich) was added, and lysates incubated at 4 °C for 2 h with rocking. The affinity matrix was pelleted by centrifugation at 2300 ***g*** for 3 min at 4 °C, washed three times in RIPA buffer, and the bound proteins eluted with the addition of SDS loading buffer.

### Plate spotting assay

10^7^ cells from exponentially growing cultures were harvested by centrifugation, and the resulting cell pellet resuspended in sterile water to achieve 10^5^ cells μL^−1^. Cell suspensions were transferred to a 96-well microtiter plate and 10-fold serially diluted to achieve 10^1^ cells μL^−1^ final. 2 μL of each dilution was spotted onto selective plates and incubated at 30 °C for 3 days before recording growth.

### mRNA extraction and cDNA synthesis

Total RNA was extracted from frozen cells using the hot acidic phenol method (Collart & Oliviero, 2001). Contaminating genomic DNA was removed by a DNase treatment step using Turbo DNA-free kit reagents as described (Applied Biosystems). End-point PCR using a forty-cycle amplification protocol and primers that flanked the intron in the *ACT1* gene was used to confirm complete removal of genomic DNA. Polyadenylated mRNA was isolated using the RNeasy kit (Qiagen) and quantified by UV spectrophotometry using the ND-2000 (NanoDrop). cDNA (125 ng of mRNA in a 20 μL reaction volume) was synthesized using the Omniscript reverse transcription kit with oligo dT primers as suggested by the manufacturer (Qiagen).

### Quantitative PCR

Oligonucleotides for real-time PCR (Table S2) were designed using the PrimeTime qPCR Assay Entry tool from Integrated DNA Technologies. The specificity of all primers was confirmed by blast analysis against the *S. cerevisiae* S288c genome. Real-time PCR was performed using 0.1 ng cDNA (in a 5 μL volume) as the template and 15 μL of DyNAmo HS SYBR Green PCR (Thermo Scientific) reagent containing gene-specific primers at the following final concentrations: 200 nM for *CIT2*, 400 nM for *ACO1*, 300 nM for *ACT1*, 100 nM for *S. cerevisiae RTG2*, and 400 nM for *A. gossypii RTG2*. Real-time PCR was performed in triplicate in a 96-well format using the StepOnePlus™ Real-Time PCR system (Applied Biosystems). Target transcripts (*CIT2*, *ACO1*, and *RTG2*) were normalized to *ACT1* levels for each strain. At least two technical replicates were performed for all samples, and data are provided for one of the two biological replicates for which data were collected. A no template control for all primer pairs was included on each plate. A standard curve, for which *rtg2Δ-Sc* cDNA was used as the template, was included on each plate for each set of primers tested for the determination of PCR efficiency. The standard curve was included in duplicate and encompassed five 10-fold dilutions of cDNA starting at 1 ng per reaction. The thermocycling program consisted of a 15-min hold at 95 °C followed by 40 cycles of 15 s at 95 °C, 1 min at 60 °C, and 30 s at 72 °C. Data from a melt curve were included after each run to verify PCR specificity and confirm the absence of PCR contamination and primer dimers. For calculations, the base of the exponential amplification function was used such that 1.95 represents a 95% amplification efficiency. Data are reported only for runs having a real-time PCR efficiency of between 85 and 110%. The differences in fold expression were normalized to *rtg2Δ-Sc* grown in the presence of glutamate and determined using the Pfaffl method (Pfaffl, 2001).

## Results

### Complementation of glutamate auxotrophy

**Fig 1:**
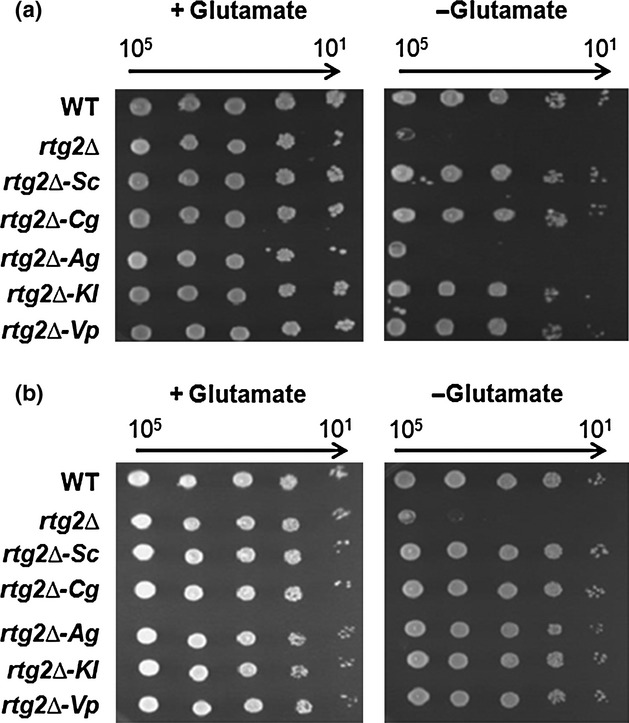
Complementation of *rtg2Δ* glutamate auxotrophy by Rtg2p homologs. 10-fold serial dilutions of cells expressing the indicated *RTG2* genes were spotted to -Trp synthetic dropout media with and without glutamate. Growth was scored after 3 days of growth at 30 °C. Complementation profiles for Rtg2p homologs expressed from the native *RTG2* promoter are shown in (a); complementation profiles for Rtg2p homologs expressed from the constitutive *GPD* promoter are shown in (b).

In cells defective for electron transport activity, the retrograde signaling pathway has been linked to cellular glutamate homeostasis through its control of the expression of the first three enzymes of the Krebs cycle (Liu & Butow, 1999). This *RTG* regulatory pathway functions to increase α-ketoglutarate production ensuring a sufficient supply of glutamate for various biosynthetic reactions. Like cells deleted for *cit1 cit2,* or *aco1*, *rtg2* deletion mutants are strict glutamate auxotrophs (Gangloff *et al*., 1990; Kim *et al*., 1986; Liao & Butow, 1993). Therefore, to determine whether the putative fungal *RTG2* homologs encode functional Rtg2p counterparts, we first tested their ability to complement the glutamate auxotrophy of *rtg2Δ*. When expressed from the endogenous *RTG2* promoter, all Rtg2p homologs complemented growth on media lacking glutamate except for *RTG2* from *A. gossypii* (Fig. 1a). Given that all homologs were expressed from the *S. cerevisiae RTG2* promoter rather than their endogenous promoters, differences in promoter recognition is not likely a source of this noncomplementation. To determine whether enhanced expression improves complementation, we retested glutamate auxotrophy using *RTG2* homologs expressed from the constitutive *GPD* promoter (Fig. 1b). Under these conditions, all Rtg2p homologs complemented the growth of *rtg2Δ,* suggesting that all homologs are functional for glutamate auxotrophy in *S. cerevisiae*. Western blot analysis confirmed the expression level of Rtg2p from *A. gossypii* was reduced relative to the levels of other Rtg2p homologs (Fig. S1). Quantitative PCR and protein half-life studies suggest that neither a reduction in mRNA levels nor increased protein turnover are likely explanations for decreased *A. gossypii* Rtg2p levels (data not shown). The potential of a translational mechanism of regulation influencing the expression of *A. gossypii* Rtg2p in the *S. cerevisiae* remains to be determined.

### Complementation of Cit2p and Aco1p levels

**Fig 2:**
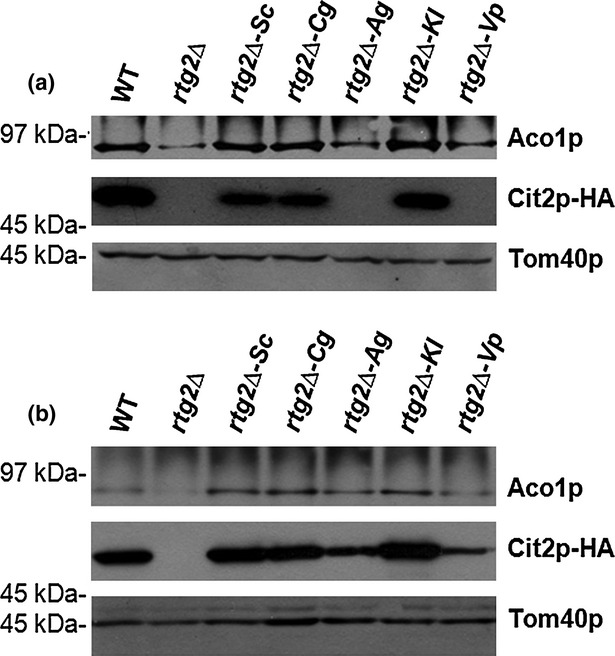
Aco1p and Cit2p protein profiles in *rtg2Δ* cells expressing Rtg2p homologs. 5 × 10^6^ cells from an exponentially growing culture supplemented with glutamate were processed by alkaline lysis followed by TCA precipitation. The resulting whole cell extracts were separated by SDS-PAGE followed by immunoblot analysis using antibodies against Aco1p, the triple-hemagglutin epitope to detect Cit2p and Tom40p. Tom40p was included as a loading control. Aco1p and Cit2p protein levels in *rtg2Δ* cells expressing Rtg2p homologs from the native *RTG2* promoter (a) and from the constitutive *GPD* promoter (b).

Activation of retrograde signaling results in the transcriptional upregulation of *CIT2,* the peroxisomal isoform of citrate synthase, and *ACO1*, an early TCA cycle enzyme. To determine the ability of *RTG2* homologs to induce the transcriptional activation of these two genes, we first determined *CIT2* and *ACO1* transcript levels under basal conditions (+ glutamate) (Table 1). Quantitative real-time PCR analysis revealed a 10- to 14-fold reduction in *CIT2* transcript levels when compared with cells expressing *RTG2* from *S. cerevisiae* for the *rtg2Δ* null mutant and for cells expressing the *RTG2* homologs from *A. gossypii* and *V. polyspora*. A threefold reduction in the level of *CIT2* transcript was found for cells expressing the *C. glabrata RTG2* homolog, while little difference was detected for cells expressing *RTG2* from *K. lactis*. Less variation was found for *ACO1* transcript levels. All *RTG2* homologs, except *V. polyspora*, had *ACO1* transcript levels that were reduced by 1- to 4-fold compared with that of *RTG2* from *S. cerevisiae*. A similar pattern was detected for Cit2p at the protein level. Cells expressing Rtg2p homologs from *C. glabrata* and *K. lactis* had levels of Cit2p and Aco1p similar to that of Rtg2p from *S. cerevisiae*. However, cells expressing *A. gossypii* and *V. polyspora* homologs showed Cit2p and Aco1p expression levels similar to that detected for *rtg2Δ* null mutants (Fig. 2a). Increasing the expression of *RTG2* homologs using the *GPD* promoter partially rescued Cit2p levels for *A. gossypii* and *V. polyspora* (Fig. 2b).

**Table 1:** Fold expression level changes for *CIT2* and *ACO1* under basal (+ glutamate) and inducing (− glutamate) conditions. mRNA was isolated from total RNA and cDNA synthesized as described in the Materials and methods. The relative expression levels of *CIT2* and *ACO1* were measured by real-time PCR using gene-specific primers. Data were normalized to *rtg2Δ-Sc* grown in the presence of glutamate and presented as a range of the fold changes obtained from several independent runs on the same biological sample

	*CIT2*		*ACO1*	
Strain	+ Glutamate	− Glutamate	+ Glutamate	− Glutamate
*rtg2*Δ	−13 to −14 (*n* = 2)	NA	−1.3 to 1 (*n* = 2)	NA
*rtg2*Δ-*Sc*	1	1.5 to 2.3 (*n* = 4)	1	−1 to −1.3 (*n* = 3)
*rtg2*Δ-*Cg*	−3 to −3.5 (*n* = 2)	−1.3 to −1.6 (*n* = 4)	−1.3 to −4.5 (*n* = 2)	−1.7 to −2.7 (*n* = 4)
*rtg2*Δ-*Ag*	−11 to −13 (*n* = 2)	NA	−0.9 to −1.3 (*n* = 2)	NA
*rtg2*Δ-*Kl*	1.1 to 1.4 (*n* = 2)	1 to 1.4 (*n* = 4)	−1.8 to −2.2 (*n* = 2)	−1.4 to −2 (*n* = 4)
*rtg2*Δ-*Vp*	−10 to −11 (*n* = 2)	−2.7 to −3.5 (*n* = 4)	1.3 to 1.6 (*n* = 2)	−2.3 to 1.5 (*n* = 4)

NA; data not available as strains were unable to grow under the indicated conditions.

**Fig 3:**
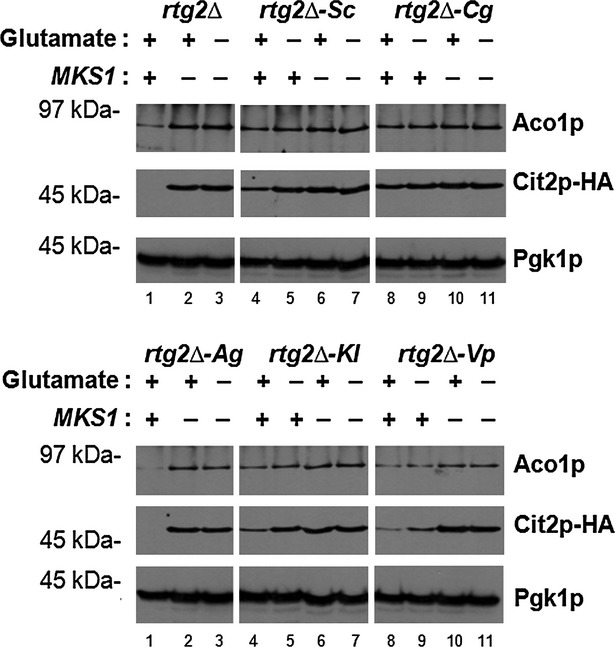
Cit2p and Aco1p protein profiles in *mks1Δ* cells expressing Rtg2p homologs. Each strain was grown in the presence or absence of glutamate as indicated. 5 × 10^6^ cells from a mid-log culture were harvested by centrifugation and whole cell extracts prepared by alkaline lysis. Total protein extracts were separated by SDS-PAGE and analyzed by immunoblotting using antibodies against Aco1p, Pgk1p, and the triple-hemagglutin epitope to detect Cit2p. Pgk1p was included as a loading control. All *RTG2* homologs were expressed from the native *RTG2* promoter.

The ability of Rtg2p homologs to activate retrograde signaling was tested by measuring *CIT2* and *ACO1* transcript levels and Cit2p and Aco1p protein levels for cells grown in the absence of glutamate, conditions known to relieve inhibition of this pathway. A modest increase in *CIT2* transcript levels over basal conditions was found for cells expressing *RTG2* from *S. cerevisiae*. A similar trend was seen for all *RTG2* homologs, except for cells expressing *RTG2* from *K. lactis*, which showed little response to induction conditions. *ACO1* transcript levels, on the other hand, showed no consistent response to inducing conditions for any of the strains tested. At the protein level, cells expressing Rtg2p homologs from *C. glabrata* and *K. lactis* showed increased expression of Cit2p and Aco1p over basal levels, similar to that detected for *S. cerevisiae* [Fig. 3; compare lanes 8–9 in (a) and lanes 4–5 in (b) with lanes 4–5 in (a)]. The increased protein levels for *K. lactis* were unexpected given the absence of a clear change at the transcript level. Although expression of Rtg2p from *V. polyspora* resulted in transcriptional activation of both Cit2p and Aco1p, the activated levels for these two proteins were lower than that of *S. cerevisiae*, consistent with the data obtained for *CIT2* by qPCR [Fig. 3; compare lane 9 in (b) with lane 5 in (a)]. Note that *CIT2* and *ACO1* transcript and protein levels could not be determined for *rtg2Δ* null cells and *rtg2Δ* cells expressing Rtg2p from *A. gossypii* as these two strains are unable to grow in the absence of glutamate. Mks1p is a negative regulator of retrograde signaling, and deletion of *MKS1* has been shown to constitutively activate the pathway, independent of *RTG2* expression (Dilova *et al*., 2002; Dilova *et al*., 2004). As expected, deletion of *MKS1* resulted in elevated levels of Cit2p and Aco1p for all strains regardless of the *RTG2* gene expressed.

### Analysis of Mks1p–Rtg2p and Mks1p–Bmh1p dynamic interaction

**Fig 4:**
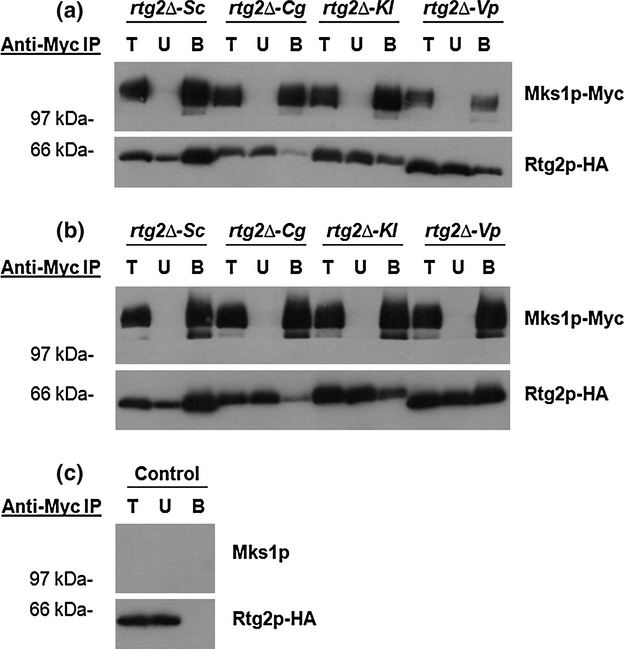
Rtg2p homologs have varied affinity for Mks1p. Immunoprecipitation of Mks1p-Myc coprecipitates Rtg2p homologs. Pull down assays were carried out on total protein lysates isolated from cells grown in the presence (a) or absence (b) of glutamate. The specificity of Mks1p–Rtg2 interaction was confirmed using cells expressing an untagged version of Mks1p (c). Cells were processed by glass bead lysis followed by immunoprecipitation of Mks1p using anti-Myc affinity beads. Total (T), unbound (U), and immunoprecipitated (B) Mks1p and the corresponding Rtg2p protein levels were detected by immunoblotting using antibodies to the Myc and HA epitopes to detect Mks1p and Rtg2p, respectively. Total and unbound samples represent 10% of the total lysate, while bound is 87% of total lysate.

The ability of fungal Rtg2p homologs to associate with the negative regulator Mks1p was tested taking a co-immunoprecipitation approach. When compared with Rtg2p from *S. cerevisiae*, Rtg2p homologs from *C. glabrata, K. lactis,* and *V. polyspora* showed a 50–80% lower affinity for Mks1p (Fig. 4a and Table S3). This reduction in Rtg2p–Mks1p interaction was seen even with induction of retrograde signaling, although the data does suggest a minor enhancement in interaction between the two proteins for all strains (Fig. 4b and Table S3). Interestingly, expression of Rtg2p from *V. polyspora* showed a similar binding affinity for Mks1p as other Rtg2p homologs despite being unable to complement Cit2p expression (Fig. 2). This suggests that Rtg2p–Mks1p interaction alone may not be sufficient for transmitting a retrograde response. We also found that a significant pool of Rtg2p was not associated with Mks1p. A role of ‘free’ Rtg2p in retrograde signaling is unclear although a number of additional functions for Rtg2p have been uncovered. For example, Rtg2p has been shown to be a component of the nuclear-localized SLIK complex, a SAGA-like histone acetyltransferase; has been implicated in suppressing the formation of extra chromosomal ribosomal circles; has been linked to genome stability through its role as a suppressor of trinucleotide repeat expansion; and has been shown to function as a molecular player in the TOR nutrient signaling pathway (Pray-Grant *et al*., 2002; Borghouts *et al*., 2004; Bhattacharyya *et al*., 2002).

**Fig 5:**
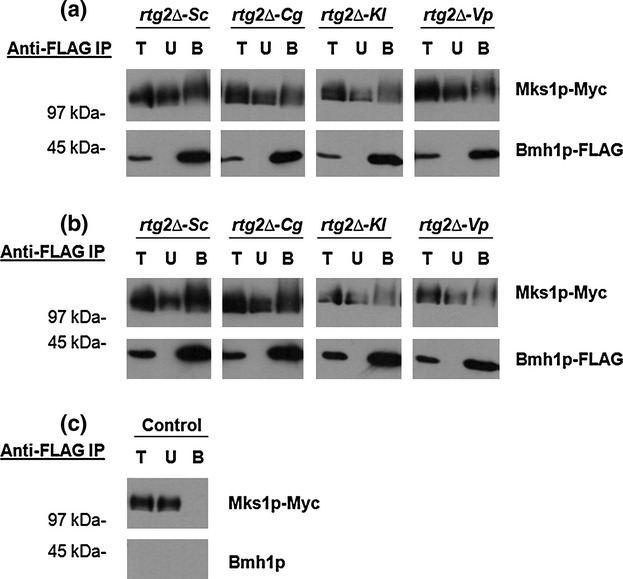
The interaction between Mks1p and Bmh1p is altered in cells expressing Rtg2p homologs. Immunoprecipitation of Bmh1p coprecipitates Mks1p. Pull down assays were carried out using cell extracts isolated from cells grown in the presence (a) or absence (b) of glutamate. The specificity of Bmh1p–Mks1p interaction was confirmed using cells expressing an untagged version of Bmh1p (c). Cells were processed by glass bead lysis followed by immunoprecipitation of Bmh1p using anti-FLAG affinity beads. Total (T), unbound (U), and immunoprecipitated (B) Bmh1p and the corresponding Mks1p protein levels were detected by immunoblotting using antibodies to the FLAG and Myc epitopes to recognize Bmh1p and Mks1p, respectively. All data were taken from the same autoradiograph under the same exposure conditions. Total and unbound samples represent 10% of the total lysates, while bound is 87% of the total lysates.

The current model for activation of retrograde signaling proposes that a dynamic interaction exists between Mks1p and Bmh1p and Mks1p and Rtg2p (Liu *et al*., 2003; Ferreira *et al*., 2005). To assess whether the decreased interaction between Mks1p and Rtg2p homologs was paralleled by an increased interaction between Mks1p and Bmh1p, a co-immunoprecipitation approach was taken. Mks1p–Bmh1p interaction was measured under both basal (+ glutamate) and inducing (- glutamate) conditions. Cells grown in the presence of glutamate showed a similar interaction affinity between Mks1p and Bmh1p irrespective of the expressed Rtg2p homolog (Fig. 5 and Table S4). Given the inverse relationship dynamics between Mks1p–Rtg2p and Mks1p–Bmh1p complex formation, cells grown under inducing conditions should decrease Mks1p–Bmh1p complex formation to facilitate an increased Mks1p–Rtg2p interaction (Liu *et al*., 2003). Interestingly, all Rtg2p homologs showed a stronger interaction between Mks1p and Bmh1p compared with that detected for *S. cerevisiae RTG2* expressing cells under inducing conditions.

## Discussion

Computational analysis identified putative Rtg2p proteins in *C. glabrata*, *A. gossypii*, *K. lactis,* and *V. polyspora*. Using an *rtg2Δ* shuffle strategy, we characterized the phenotypes associated with the expression of these fungal Rtg2p homologs in the model yeast *S. cerevisiae*.

Our data show that these four fungal Rtg2p homologs are functional in transmitting a signal from dysfunctional mitochondria to the nucleus and in inducing a cellular response. *Candida glabrata* and *K. lactis* Rtg2p homologs were fully functional with regard to all cellular responses tested. The *V. polyspora* Rtg2p homolog was able to rescue glutamate auxotrophy but showed reduced transcriptional activation of *CIT2* and an equivalent decrease in Cit2p levels, while the functional activity of the *A. gossypii* Rtg2 homolog was minimal due to poor expression in *S. cerevisiae*. A similar expression pattern was detected for aconitase protein in these strains, although the *ACO1* transcript level data were less obvious. The absence of a direct correlation between Rtg2 homolog functionality and *ACO1* transcription may reflect the fact that *ACO1* transcription is under the control of both the *HAP* regulatory system and the *RTG* response pathway (Liu & Butow, 1999). Unlike *CIT2,* which requires a functional *RTG* system for expression, *ACO1* transcription is dependent on the *RTG* pathway only in respiratory-deficient cells. Overall, these data suggest that the four fungal species have a molecular retrograde signaling mechanism similar to that found in *S. cerevisiae*.

We also analyzed the molecular interaction between Rtg2p homologs and Mks1p. Our results showed that all fungal Rtg2p homologs had reduced binding to Mks1p compared with that of *S. cerevisiae* Rtg2p. However, they all showed a minor increase in Mks1p interaction under inducing conditions. In addition, expression of Rtg2p homologs resulted in an apparent increase in interaction between Mks1p and Bmh1p when retrograde signaling was activated. This suggests that Rtg2p may have a role in influencing Mks1p–Bmh1p interaction. It is possible that an interaction between Rtg2p and Mks1p is required for promoting a molecular change in Mks1p that functions to prevent its inhibitory effects on Rtg1p/Rtg3p thereby activating the pathway.

Single amino acid mutations that eliminate Rtg2p activity have been generated within the ATP binding domain of Rtg2p and include Gly154Asp, Gly266Val, and Gly311Asp (Liu *et al*., 2003). Comparing amino acid sequences for Rtg2p homologs reveal sequence conservation for amino acids at positions 154 and 266, but not 311 (Fig. S2). At position 311, the *RTG2* homolog from *C. glabrata* codes for an alanine instead of glycine. For the cellular defects tested (glutamate auxotrophy, Cit2p and Aco1p protein levels), the *C. glabrata* homolog fully complemented the *S. cerevisiae rtg2Δ* strain, suggesting that conservative amino acid changes at this position are likely tolerated with minimal, if any, impact on Rtg2p activity. Targeted mutagenesis approach would confirm this hypothesis.

As a key player in the retrograde response pathway, Rtg2p has been shown to function in multiple biological processes including yeast life span, nutrient signaling, genome stability, and in metabolism as a transcriptional co-activator. We propose that the Rtg2p homologs characterized in this study are useful tools for identifying the amino acid domains that may be required for these different activities. For example, the *V. polyspora RTG2* homolog complemented glutamate auxotrophy and exhibited moderate affinity for Mks1p binding but was significantly deficient in promoting the transcriptional activation of *CIT2*. Studies by Liu *et al*., (2003) had suggested that amino acids within the ATP binding domain of Rtg2p were essential for Mks1p interaction, and in turn, *CIT2* expression. Our results using the *V. polyspora RTG2* homolog suggests that propagation of the retrograde signal requires more than a physical interaction between Rtg2p and Mks1p. As suggested by others, nucleotide binding and/or hydrolysis by Rtg2p may function to modify Mks1p, which in turn relieves Mks1p inhibition on retrograde signaling (Liu *et al*., 2003, 2005). This activity may be absent for the *V. polyspora RTG2* homolog when expressed in the *S. cerevisiae* cellular background. Future studies aimed at monitoring the phosphorylation status of Mks1p in this strain may prove insightful.

## Statement

This work was completed in partial fulfillment of the requirements for a Ph.D. (ESU).

## Supplementary material

**Fig. S1.** Comparison of steady state protein levels for Rtg2p homologs expressed from the RTG2 promoter. **Fig. S2.** Regions of amino acid similarity for Rtg2p homologs. **Table S1.** Plasmids generated for this study. **Table S2.** Gene specific primers used for detection of genomic DNA contamination and for quantitative realtime PCR. **Table S3.** Relative affinity of Rtg2p homologs for Mks1p. **Table S4.** Relative affinity of Bmh1p for Mks1p in cells expressing Rtg2p homologs.
