# Supplementary material for: Characterization of fungal RTG2 genes in retrograde signaling of Saccharomyces cerevisiae
Source: FEMS Yeast Res. 2013 Jun 20;13(5):495–503. doi: 10.1111/1567-1364.12055 (PMC3814403; doi:10.1111/1567-1364.12055)
Supplement: Supplementary file 1 — Fig. S1. Comparison of steady state protein levels for Rtg2p homologs expressed from the RTG2 promoter. Fig. S2. Regions of amino acid similarity for Rtg2p homologs. Table S1. Plasmids generated for this study. Table S2. Gene specific primers used for detection of genomic DNA contamination and for quantitative realtime PCR. Table S3. Relative affinity of Rtg2p homologs for Mks1p. Table S4. Relative affinity of Bmh1p for Mks1p in cells expressing Rtg2p homologs. [file fyr0013-0495-sd1.doc]

Supplemental material

**Fig. S1.** Comparison of steady state protein levels for Rtg2p homologs expressed from the *RTG2* promoter. Whole cell protein extracts were prepared by alkaline lysis from 5x10^6^ cells grown at 30˚C in -Tryptophan synthetic drop out media. Protein samples were separated by SDS-PAGE followed by immunoblot analysis using antibodies against the HA epitope to detect Rtg2p. Pgk1p protein levels were used to confirm equal loading.

**Fig. S2.** Regions of amino acid similarity for Rtg2p homologs. Amino acid alignment was carried out using the T-Coffee Alignment Tool.

**Table S1.** Plasmids generated for this study.

**Table S2.** Gene specific primers used for detection of genomic DNA contamination and for quantitative real-time PCR.

**Table S3.** Relative affinity of Rtg2p homologs for Mks1p. Autoradiographs were scanned and images were analyzed using NIH Image J software. Ratios were calculated using the intensity of protein bands from the same autoradiograph. For each strain, fold changes were calculated by dividing the ratio of Rtg2p/Mks1p by the ratio of Rtg2p/Mks1p interaction of *rtg2Δ* expressing Rtg2p from *S. cerevisiae*. Fold changes were calculated separately for basal (+ Glutamate) and inducing (- Glutamate) conditions. Data was collected from 3 independent experiments.

**Table S4.** Relative affinity of Bmh1p for Mks1p in cells expressing Rtg2p homologs. Autoradiographs were scanned and images analyzed using NIH Image J software. Ratios were calculated using the protein bands from the same autoradiograph. For each strain, fold changes were calculated by dividing the ratio of Mks1p/Bmh1p by the ratio of Mks1p/Bmh1p of *rtg2Δ* expressing Rtg2p from *S. cerevisiae*. Fold changes were calculated separately for basal (+ Glutamate) and inducing (- Glutamate) conditions. Data was collected from 3 independent experiments.

Figure S1.


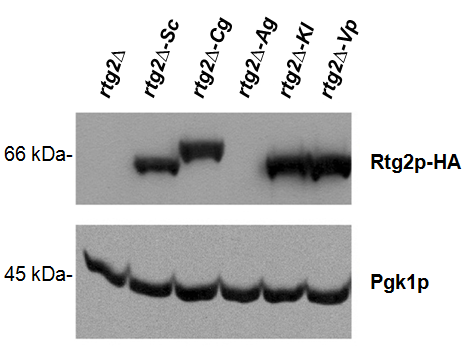


Figure S2.

Table S1.

| Plasmids | Description | Reference |
| --- | --- | --- |
| pDG54 | pFA6a-*KanMX6* | Longtine *et al*., 1998 |
| pDG62 | pFA6a-3XHA*-HIS3MX6* | Longtine *et al*., 1998 |
| pDG264 | pFA6a-3XFlag-*HIS3MX6* | This Study |
| pDG49 | YCplac22-*GPD_promoter_* (2µ, Amp^r^, *TRP1*) |  |
| pDG97 | pRS414 (*CEN/ARS, Amp^r^, TRP1*) | Sikorski *et al*, 1991 |
| pDG98 | pRS416 (*CEN/ARS, Amp^r^, URA3*) | Sikorski *et al*, 1991 |
| pDG127 | pRS416-*RTG2_promoter_* (*RTG2*, *CEN/ARS, Amp^r^, URA3*) | This Study |
| pDG176 | YCplac22-*GPD_promoter_* (*RTG2_S. cerevisiae_,* 2µ*, Amp^r^, TRP1*) | This Study |
| pDG177 | YCplac22-*GPD_promoter_* (*RTG2_C. glabrata_,* 2µ*, Amp^r^, TRP1*) | This Study |
| pDG178 | YCplac22-*GPD_promoter_* (*RTG2_V. polyspora_,* 2µ*, Amp^r^, TRP1*) | This Study |
| pDG184 | YCplac22-*GPD_promoter_* (*RTG2_K. lactis_,* 2µ*, Amp^r^, TRP1*) | This Study |
| pDG194 | YCplac22-*GPD_promoter_* (*RTG2_A. gossypii_,* 2µ*, Amp^r^, TRP1*) | This Study |
| pDG193 | pRS414 –*RTG2_promoter_* (*Cen/Ars, Amp^r^, TRP1*) | This Study |
| pDG198 | pRS414-*RTG2_promoter_* (*RTG2_S. cerevisiae_, CEN/ARS, Amp^r^, TRP1*) | This Study |
| pDG199 | pRS414-*RTG2_promoter_* (*RTG2_C. glabrata_, CEN/ARS, Amp^r^, TRP1*) | This Study |
| pDG195 | pRS414-*RTG2_promoter_* (*RTG2_A. gossypii_, CEN/ARS, Amp^r^, TRP1*) | This Study |
| pDG196 | pRS414-*RTG2_promoter_* (*RTG2_K. lactis_, CEN/ARS, Amp^r^, TRP1*) | This Study |
| pDG197 | pRS414-*RTG2_promoter_* (*RTG2_V. polyspora_, CEN/ARS, Amp^r^, TRP1*) | This Study |
| pDG246 | pRS414-*RTG2_promoter_* (*RTG2_S. cerevisiae_-3XHA, CEN/ARS, Amp^r^, TRP1*) | This Study |
| pDG229 | pRS414-*RTG2_promoter_* (*RTG2_C. glabrata_-3XHA, CEN/ARS, Amp^r^, TRP1*) | This Study |
| pDG232 | pRS414-*RTG2_promoter_* (*RTG2_A. gossypii_-3XHA, CEN/ARS, Amp^r^, TRP1*) | This Study |
| pDG250 | pRS414-*RTG2_promoter_* (*RTG2_K. lactis_-3XHA, CEN/ARS, Amp^r^, TRP1*) | This Study |
| pDG227 | pRS414-*RTG2_promoter_* (*RTG2_V. polyspora_-3XHA, CEN/ARS, Amp^r^, TRP1*) | This Study |

Table S2.

| **Primers** | **Target gene** | **Sequence (5’ to 3’)** |
| --- | --- | --- |
| For gDNA detection |  |  |
| ACT1 gDNA-F | *ACT1* | CTGAATTAACAATGGATTC |
| ACT1 gDNA-R | *ACT1* | GAGCTTCATCACCAACGTAG |
|  |  |  |
| For real-time PCR |  |  |
| ACT1 qPCR-F | *ACT1* | TCGAACAAGAAATGCAAACCG |
| ACT1 qPCR-R | *ACT1* | GGCAGATTCCAAACCCAAAAC |
| CIT2 qPCR-F | *CIT2* | CGGTATTCGTTTCAGAGGTCG |
| CIT2 qPCR-R | *CIT2* | GCTTCTGGTAGTGGTTGTGAG |
| ACO1 qPCR-F | *ACO1* | AGGACAAGGATGGTAATGAGTTC |
| ACO1 qPCR-R | *ACO1* | ACGGTCTGAAGTTGGAGAAAC |

Table S3.

| **Ratio** | **Glu** | **Strains** | | | |
| --- | --- | --- | --- | --- | --- |
|  |  | ***Δrtg2-Sc*** | ***Δrtg2-Cg*** | ***Δrtg2-Kl*** | ***Δrtg2-Vp*** |
| **Bound Rtg2p**  **Total Rtg2p** | **+** | 0.19± 0.01 | 0.024 ± 0.04 | 0.033 ± 0.01 | 0.051 ± 0.01 |
| **Bound Rtg2p**  **Bound Mks1p** | **+** | 0.70 ± 0.01 | 0.11 ± 0.03 | 0.33 ± 0.01 | 0.26 ± 0.01 |
|  | **Fold Change** | 1.00 | 0.16 | 0.47 | 0.37 |
| **Bound Rtg2p**  **Total Rtg2p** | **-** | 0.18 ± 0.01 | 0.032 ± 0.05 | 0.068 ± 0.04 | 0.085 ± 0.01 |
| **Bound Rtg2p**  **Bound Mks1p** | **-** | 1.32 ± 0.03 | 0.28 ± 0.04 | 0.68 ± 0.04 | 0.57 ± 0.04 |
|  | **Fold Change** | 1.00 | 0.21 | 0.52 | 0.43 |

Table S4.

| **Ratio** | **Glu** | **Strains** | | | |
| --- | --- | --- | --- | --- | --- |
|  |  | ***Δrtg2-Sc*** | ***Δrtg2-Cg*** | ***Δrtg2-Kl*** | ***Δrtg2-Vp*** |
| **Bound Mks1p**  **Total Mks1p** | **+** | 0.07 ± 0.01 | 0.06 ± 0.01 | 0.07 ± 0.01 | 0.07 ± 0.01 |
| **Bound Mks1p**  **Bound Bmh1p** | **+** | 0.59 ± 0.02 | 0.59 ± 0.08 | 0.62 ± 0.01 | 0.57 ± 0.01 |
|  | **Fold Change** | 1.00 | 1.00 | 1.05 | 0.97 |
| **Bound Mks1p**  **Total Mks1p** | **-** | 0.09 ± 0.01 | 0.08 ± 0.01 | 0.08 ± 0.01 | 0.06 ± 0.01 |
| **Bound Mks1p**  **Bound Bmh1p** | **-** | 0.15 ± 0.01 | 0.25 ± 0.02 | 0.25 ± 0.03 | 0.25 ± 0.02 |
|  | **Fold Change** | 1.00 | 1.67 | 1.67 | 1.67 |
